# Does the COVID-19 Vaccine Still Work That “Most of the Confirmed Cases Had Been Vaccinated”? A Content Analysis of Vaccine Effectiveness Discussion on Sina Weibo during the Outbreak of COVID-19 in Nanjing

**DOI:** 10.3390/ijerph19010241

**Published:** 2021-12-26

**Authors:** Hao Gao, Qingting Zhao, Chuanlin Ning, Difan Guo, Jing Wu, Lina Li

**Affiliations:** 1 School of Journalism and Communication, Nanjing Normal University, Nanjing 210097, China; 42396@njnu.edu.cn (H.G.); huiguaiwandepi@163.com (Q.Z.); guodifan@163.com (D.G.); 2 School of Media and Communication, Shanghai Jiao Tong University, Shanghai 200240, China; ningchuanlin@sjtu.edu.cn; 3 Faculty of Social Sciences, University of Ljubljana, 1000 Ljubljana, Slovenia; sunnyjingya@163.com; 4 Film-Television and Communication College, Shanghai Normal University, Shanghai 200234, China

**Keywords:** breakthrough cases, COVID-19 vaccine, sentiment orientation, social media

## Abstract

In July 2021, breakthrough cases were reported in the outbreak of COVID-19 in Nanjing, sparking concern and discussion about the vaccine’s effectiveness and becoming a trending topic on Sina Weibo. In order to explore public attitudes towards the COVID-19 vaccine and their emotional orientations, we collected 1542 posts under the trending topic through data mining. We set up four categories of attitudes towards COVID-19 vaccines, and used a big data analysis tool to code and manually checked the coding results to complete the content analysis. The results showed that 45.14% of the Weibo posts (*n* = 1542) supported the COVID-19 vaccine, 12.97% were neutral, and 7.26% were doubtful, which indicated that the public did not question the vaccine’s effectiveness due to the breakthrough cases in Nanjing. There were 66.47% posts that reflected significant negative emotions. Among these, 50.44% of posts with negative emotions were directed towards the media, 25.07% towards the posting users, and 11.51% towards the public, which indicated that the negative emotions were not directed towards the COVID-19 vaccine. External sources outside the vaccine might cause vaccine hesitancy. Public opinions expressed in online media reflect the public’s cognition and attitude towards vaccines and their core needs in terms of information. Therefore, online public opinion monitoring could be an essential way to understand the opinions and attitudes towards public health issues.

## 1. Introduction and Literature Review

### 1.1. The Breakthrough Cases in the Nanjing Outbreak and COVID-19 Vaccine Hesitancy

China has implemented strict measures to respond to the epidemic since the outbreak of COVID-19, which enabled the epidemic to be placed under control [1]. In addition to sporadic imported cases, indigenous infections occasionally occur and these can be cleared within 2–3 incubation periods [2]. On 20 July 2021, nine confirmed cases were detected at Nanjing Lukou International Airport during routine nucleic acid tests on staff [3], which triggered a regional outbreak of COVID-19 in Nanjing, the capital city of East China’s Jiangsu Province. The epidemic then spread from Nanjing to other cities of China rapidly as the airport was the origin of the confirmed cases. The latest outbreak in Nanjing has spread to 16 provinces, and over 400 infections have been reported up to 3 August 2021 [4]. The new surge of COVID-19 cases starting in Nanjing and spreading nationwide have attracted public attention.

To better control the epidemic, Mainland China has provided COVID-19 vaccines free of charge to the public since January 2021 [5]. Since then, China has been promoting COVID-19 vaccination nationwide, which includes educating and providing vaccine knowledge, emphasizing the necessity of being vaccinated, and disseminating information on the effectiveness of vaccines. China has accomplished great vaccination coverage, with more than 1.7 billion doses having been administered (two doses per person) as of 4 August 2021 [6]. Hence, members of the public have expressed concerns about whether the confirmed cases in the Nanjing outbreak had been vaccinated before. On 23 July 2021, the Nanjing municipal government announced that 17 out of 18 confirmed cases had been vaccinated except one minor. As most of the confirmed cases had mild symptoms, medical experts reiterated the effectiveness of vaccines and the importance of being vaccinated at the press conference [7]. The occurrence of breakthrough cases in the Nanjing outbreak surprised the public. On Sina Weibo, the largest social media platform in China, people discussed and expressed their opinions on the effectiveness of the COVID-19 vaccine, with a hot issue tagged as #*most of the confirmed cases in Nanjing had been vaccinated*.

The public has shown fear and hesitancy in relation to various types of vaccines, including COVID-19 [8]. Due to the urgent need for controlling the pandemic, the development, production, and mass roll-out of COVID-19 vaccines progressed faster than that of any existing vaccine, which caused public concern about the effectiveness and safety of the vaccine [9,10]. Some researchers have developed and used metrics and scales to measure vaccine confidence and hesitancy [11]. In particular, the Vaccine Confidence Index (VCI) measures the perceptions of individuals regarding the safety, importance, and effectiveness of vaccines as a core indicator of public confidence in vaccines [12]. Furthermore, studies have found that a lack of confidence in the vaccine will be a barrier to achieving herd immunity, which is essential to ending the epidemic [13]. Multiple surveys on COVID-19 vaccine trust and hesitancy during the pandemic have indicated the significance and necessity of regularly monitoring public opinions toward the effectiveness and safety of COVID-19 vaccines [14]. Tracking public perceptions of COVID-19 vaccines helps policymakers to better understand the reasons behind vaccine hesitancy and how to better implement vaccine roll-out strategies [14].

### 1.2. Social Media Discourse and Attitudes towards Vaccines

Social media has been a primary means of sharing and seeking health-related information, where people freely generate information, leave comments, express opinions, and communicate with others on specific topics [15,16]. Information about the COVID-19 vaccines triggered a great deal of public interest and response on social media [17], providing an opportunity to understand public opinion about COVID-19 vaccines [18]. Public attitudes to vaccines can be reflected by posts and comments on social media [19]. A study on Twitter posts related to COVID-19 vaccines conducted a thematic analysis of posts marked as negative sentiments and discovered that concerns about the safety and effectiveness of vaccines ranked second among all themes [13].

Furthermore, individuals’ attitudes towards vaccines are largely driven by online information and opinions posted on social media, especially by negative information [20,21,22]. Studies have shown that exposure to negative emotions regarding vaccination on social media exacerbated vaccine hesitancy and refusal [13]. Several studies on social media and attitudes towards vaccines have also shared the same findings that exposure to misinformation, rumors, and negative emotions vaccination on Twitter increased vaccine hesitancy and refusal, even causing a decline in the vaccination rate [23,24,25]. Discussion about COVID-19 vaccines on social media provides a source of data, and the knowledge of these discussions can explain users’ attitudes, acceptance, or hesitancy towards COVID-19 vaccines [8]. It is cost-effective to investigate public opinions through social media, unraveling more pluralized and authentic perceptions than traditional public opinion surveys [26].

### 1.3. Social Media Discourse and Sentiment Analysis

As mentioned above, social media analysis provides insights into public attitudes and hesitancy towards vaccination [22]. In terms of the research methods, social media analysis often extracts data via big data, further analyzing the data through sentiment analysis and thematic analysis tools. A large number of previous studies have experimented with methods aiming to understand the public discourse around COVID-19 and investigate topics related to the pandemic, such as qualitative content analysis [27], sentiment analysis, word frequency analysis [28], and topic modeling [29,30,31]. These studies extracted topics and sentiments in social media discussions related to the COVID-19 vaccines and identified the changes in topics and sentiments over time to better understand the public opinions, concerns, and emotions [8,32].

Sentiment analysis is particularly significant in vaccine research now that anti-vaccine emotions have been prevalent online for a long time, especially in Europe and the US [33]. However, anti-vaccine information on social media from different cultures and national contexts might differ. Stella et al. [34] investigated the public emotions and social repercussions during the lockdown in Italy, the first country with a national lockdown due to the pandemic. They found complex emotions in which anger and fear coexisted with trust, solidarity, and hope. Another study comparing American and Chinese posts on Twitter and Sina Weibo revealed differences in public perceptions of COVID-19 among people in different cultures [35].

Sentiment analysis is an important method for extracting and analyzing public opinions and views, with a considerable number of established analytical tools. Methods and tools for sentiment analysis mainly include two categories: lexicon-based and machine learning-based classifications [36]. In terms of the accuracy in sentiment analysis, even the commonly used dictionaries cannot completely identify the sentiment tendencies of texts [37], but manually built sentiment lexicons are more accurate than others [38]. However, lexicons and dictionary development is time-consuming, staff intensive, and costly [39]. Hence, most studies have continued to adopt the commonly used dictionary and lexicons for sentiment analysis.

Words drawn from the general-domain lexicons cannot handle the sentiments of all domains [40], which limits the accuracy of the sentiment dictionary approach to a certain extent. Moreover, the meaning of words on social media may change with the contextual environment. Furthermore, the extraction of positive or negative sentiments is generally based on polarity orientation but without reference to specific contexts. On this occasion, capturing accurate attitudinal tendencies requires a detailed analysis of content based on sentiment analysis, especially combined with the contextual environment [41]. In this study we used content analysis for the manual determination of attitudes to make up for the deficiency of sentiment analysis tools.

Under the context of the breakthrough cases occurring in the Nanjing outbreak, this study aimed to explore public perceptions of vaccine effectiveness and examine whether this wave of the epidemic influenced vaccine hesitancy and distrust. Thus, this study conducted content analysis on Sina Weibo posts with the tag *#most of the confirmed cases in Nanjing had been vaccinated*, proposing the following research questions:

RQ1. Did the comments of social media users generally indicate negative attitudes towards COVID-19 vaccines?

RQ2. Did these comments clearly express negative emotions or sentiments?

RQ3. What are the insights behind the expression of attitudes and sentiments towards COVID-19 vaccines?

## 2. Materials and Methods

### 2.1. Research Sampling

A hot issue tagged as *#most of the confirmed cases in Nanjing had been vaccinated* gained public attention after the Nanjing municipal government released the same news. Then this hashtag became the trending topic on Weibo on 23 July 2021, with a total of 135 min on the Sina Weibo Hot Search List (HSL) and a Hot Search Index of 3,258,154 [42]. The number of posts with the hashtag reached 6221 on 23 July alone, which shows great concern and public participation in relation to the issue. Hence, we selected the posts with this hashtag as the research sample.

The topic was heating up for 5 to 6 days, and the number of posts under the topic tended to level off after that point. So, we targeted the period within the most discussion as the research sample, from 23 to 26 July. We used self-written Python scripts to connect to the API of Sina Weibo and collected 6883 posts under the topic. Then, we manually removed posts with emojis; pictures; videos; and repeated, irrelevant, advertising, or abnormal information. Finally, we obtained a sample with 1542 valid posts.

### 2.2. Data Processing

This paper utilized DivoMiner^®^ for online content analysis, developed by Zhuhai (China) Hengqin Boyi Data Technology Co., LTD (Zhuhai, China). This tool combines traditional content analysis with big data analysis, allowing for the creation of sample databases, the setting of categories for code books, intrinsic coder reliability testing, formal coding, quality monitoring, statistical analysis, and the visualization of results. Research on health communication, international communication, and management science has widely utilized the tool, as proven in many published journals [43]. We also conducted a content analysis of the selected posts with this tool.

### 2.3. Category Setting

Although previous studies have provided valuable insights into the COVID-19 vaccines, there are limitations in their evaluation of public attitudes towards vaccines through sentiment analysis. Most research related to online sentiments focuses on sentiment polarity, which divides sentiments into positive, negative, and neutral [44]. Simple categorical sentiment analysis ignores the subtle sentimental changes of users and cannot reflect the complex mental world. Hence, a fine-grained sentiment analysis based on multiple categories is necessary for examining sentiment attribution [45]. Moreover, the sentiments presented in online posts are complex. It is of significance to consider both sentimental appearances and sentiment targets. Sentiment analysis is targeted at the lexical sentiment orientation and cannot completely explain the public attitudes due to different contexts. Thus, in this study we used manual coding to determine attitudes based on previous experience.

In terms of specific coding, for this study we established four categories. The first category is attitudes to vaccines because the focus of this study is about the public attitudes to the COVID-19 vaccines. Considering that there are contextual differences in online texts, we need to consider the specific targets when evaluating the textual attitude tendencies. Thus, the second category refers to the specific targets of different sentiment orientations. The other two categories are common indicators for sentiment analysis, namely, sentiment polarity and sentiment attribution.

As to the evaluation of attitudes towards COVID-19 vaccines, in this study we classified attitudes into four types: supportive, neutral, doubtful, and undetermined. The content would be considered ‘supportive’ in cases in which the user clearly expressed a positive opinion of the vaccine and support for vaccination. If the user questioned the vaccine’s effectiveness, the post would be defined as ‘doubtful’. If the user did not express support or doubt when talking about COVID-19 vaccines, the post would be judged to be ‘neutral’. ‘Undetermined’ content referred to posts unrelated to the vaccine, or in which the user’s attitude to vaccines could not be determined through the content.

Sentiment polarity and sentiment attribution were coded through a man-machine combination approach in this study. Referencing the DLUT-Emotion ontology [46], a mainstream sentiment analysis tool in China, sentiment polarity was classified into three categories: positive, neutral, and negative, and sentiment attribution was classified into eight dimensions: good, happy, surprise, disgust, sadness, fear, anger, and other. In terms of operation, we introduced the dictionary of DLUT-Emotion ontology in the first step. There are more than 20,000 commonly used Chinese sentiment words in the dictionary, with each word labeled with sentiment polarity and sentiment attribution. Secondly, the computer matched the words contained in the posts with words in the dictionary, then determined each text’s sentiment polarity and attribution. However, the tool is based on word-by-word analysis and cannot take the contextual meaning of sentences into account during the analysis. Considering that the results might be biased on this occasion, we checked and corrected the results manually in the third step. Specifically, we referred to DLUT-Emotion ontology to annotate the sentiment words contained within the sample. Then, we synthesized the annotation results and sentence meaning for sentiment coding. Table 1 shows the examples of sentiment words and the sentiment coding of comments.

Finally, we determined the specific targets of different sentiment orientations, including COVID-19 vaccines, epidemic/novel coronavirus, media, government/state, posting user, the public, and undetermined. Table 2 shows the coding items and rules of the content analysis.

### 2.4. Coding Reliability

We constructed an online reliability test bank with a 10% randomly selected sample at DivoMiner^®^ for independent coding by three coders. Then, we matched the results of the three coders by pairs, using the Holsti coefficient as the indicator for reliability calculations [47]. Through multiple corrections of the coding criteria and the combination of reliability indices for each variable, the final composite reliability of the three coders was 0.82, which reached the standard [48].

## 3. Results

### 3.1. Attitudes towards the COVID-19 Vaccine

Table 3 shows the result of the public attitudes to COVID-19 vaccines according to the Weibo posts with the tag #*most of the confirmed cases in Nanjing had been vaccinated*. We found that 7.3 percent of posts showed doubtful attitudes (*n* = 112), 13 percent of posts indicated neutral attitudes (*n* = 200), 45 percent of posts were supportive (*n* = 696), and undetermined attitudes accounted for 34.6 percent of posts (*n* = 534). In general, the public showed significant concerns with the COVID-19 vaccine due to breakthrough cases in Nanjing. Nevertheless, they did not question the vaccine’s effectiveness intensely during this wave of the epidemic.

### 3.2. Sentiment Analysis

Although Weibo users did not question the effectiveness of the COVID-19 vaccine intensely, Table 3 indicates significant negative sentiment polarity and attributes. Posts judged as negative emotions accounted for 66.47% (*n* = 1025) of posts, whereas positive emotions only accounted for 12.97% (*n* = 200). Furthermore, the dominant sentiments shown in these posts were disgust (39.50%, *n* = 609), anger (16.86%, *n* = 260), and others (25.88%, *n* = 399). Only 11.15% (*n* = 172) reflected a positive sentiment, corresponding to the ‘good’ attribute.

### 3.3. Correlation Analysis of Sentiment Orientations and Attitudes towards COVID-19 Vaccines

As shown in Table 4, there was a correlation between sentiment orientations and attitudes towards COVID-19 vaccines. So, did the posts defined as expressing negative emotions (66.47%) reflect vaccine hesitancy or distrust? Table 3 shows that over half of the 1025 posts with negative emotions (*n* = 517, 50.44%) were directed towards the media. The second and third most frequent targets of negative emotions were posting users (*n* = 257, 25.07%) and the public (*n* = 118, 11.51%). In addition, the specific targets of ‘disgust’ and ‘anger’ were the same as those of negative emotions. Among the 609 posts of ‘disgust’, 334 posts (54.84%) pointed to the media, 189 posts (31.03%) pointed to posting users, and 66 posts (10.84%) were directed at the public.

Table 3 also indicates the cardinal interaction results of Sina Weibo users’ emotional orientations and emotional targets, with 397 posts explicitly expressing emotional orientations towards vaccines. Among them, 178 posts (44.83%) are positive, 150 posts (47.32%) are neutral, and only 69 posts (6.73%) are negative. Moreover, 178 out of 200 positive posts were directed at the vaccine.

Overall, the discussion on the tag *#most of the confirmed cases in Nanjing had been vaccinated* showed that the public mainly still supported the COVID-19 vaccine, rather than questioned its effectiveness. Although the public expressed strong negative emotions in their discussion, they were directed at the media, posting users, and the public, rather than at the COVID-19 vaccine.

## 4. Discussion

### 4.1. Why Did the Breakthrough Cases in Nanjing Not Trigger Strong Doubt about the Effectiveness of the COVID-19 Vaccine?

The occurrence of breakthrough cases in Nanjing has not caused strong doubt about the effectiveness of the COVID-19 vaccine. According to the content analysis of attitudes of Weibo users towards COVID-19 vaccines, only 7.26% (*n* = 112) of posts expressed doubts about the effectiveness of the vaccines and opposed vaccination, except for 34.63% of posts, for which we were unable to determine the attitude tendency. The remaining 45.14% had positive attitudes of support and trust in COVID-19 vaccines, and 12.97% were neutral to the vaccines. Another study examining the differences in attitudes towards vaccines between the US and China through social media discussions shared the same finding. Compared to a large number of anti-vaccination discussions on Twitter, similar discourse is rare on Sina Weibo [47]. Previous studies maintained that pro-vaccine information usually revolves around potential side effects, adverse vaccine reactions, misinformation, and conspiracy theories [35]. Apart from conspiracy theories, anti-vaccination discussions among the Chinese public showed similar topics. Users who questioned the efficacy clearly expressed that ‘the vaccine is ineffective as the confirmed cases had been vaccinated’. Others questioned the view proposed by the media and medical experts that ‘vaccines can reduce the probability of severe infection’. They argued that the current evidence could not prove the direct relation between vaccination and a low incidence of severe infection. In addition, a few users questioned whether the ADE effect of the domestic vaccine occurred as most of the confirmed cases had been vaccinated. People generally questioned the vaccine mainly because of ‘the inadequacy of current data and evidence on vaccine effectiveness’ and ‘the lack of objectivity in media reports’.

These findings are in line with previous research, in which a positive tone dominated Weibo posts compared to Twitter posts in the US [47]. The fact that 45.14% of posts were marked as positive attitudes showed that the public was still optimistic about the COVID-19 vaccine and expected the vaccine to defeat the pandemic. Some users directly acknowledged the effectiveness of the COVID-19 vaccine and believed that the vaccine had played a significant role in controlling the epidemic. For example, they wrote, ‘if the vaccines had not been administered, more people might have been infected’ and ‘people who were not infected might have been protected by the vaccine’, expressing their recognition and knowledge of its effectiveness. Furthermore, some users maintained that the vaccine could not completely protect people against the virus, especially when facing new variants of the virus, but they still recognized that vaccination can reduce the probability of severe infections. They often quoted views of professionals or media releases, which indicated that they agree with these opinions. Additionally, although some users did not specify the vaccine’s effectiveness, they believed that vaccination was more helpful in building a protection mechanism than a situation without vaccination. They used metaphors to articulate their understanding of the vaccine. For example, they wrote’, being vaccinated is like putting a toughened film on your mobile phone’, and ‘clothing is much better than naked’. Overall, these posts showed the positive perceptions and attitudes of the posting users towards vaccine effectiveness, which were derived from the knowledge of the COVID-19 vaccine [49]. Their knowledge originated from education and communication when promoting vaccination [50], resulting from the previous vaccination promotion. People gained more knowledge and a more positive perception of the vaccine after the previous large-scale vaccination promotion, which further influenced their attitudes towards the vaccine.

Furthermore, the content of posts marked as neutral attitudes towards the vaccine mainly focused on four aspects, which were all related to the knowledge of the vaccine and epidemic prevention. First, these users suggested wearing masks and washing hands regularly even if they had been vaccinated. Second, some users explained the effect mechanisms and other knowledge of the vaccine with objective words. Third, some users posed questions about whether the COVID-19 vaccine can protect people against variants of the virus. Fourth, special groups, such as pregnant women and people with chronic diseases, inquired whether they could be vaccinated. This content indicates a high level of public knowledge about the vaccine and also indicates that knowledge predicts a positive attitude to the vaccine [51].

In addition, we found that Weibo users were prone to showing evident respect for the authorities and a more positive attitude towards COVID-19 vaccines. In comparison, Twitter users tended to share personal vaccination experiences and expressed anti-vaccination attitudes [47]. The Chinese public presented a more collectivist cultural identity than the individualistic cultural traits of the US.

### 4.2. Why Did These Posts Present Strong Negative Emotions from Users?

In contrast to the dominance of positive and neutral attitudes towards COVID-19 vaccines, sentiment analysis found significant negative emotions and sentiments in Weibo posts. This is intriguing as previous studies proved that sentiment analysis provides insight into public attitudes towards vaccines [22]. However, this study indicated no correlation between public attitudes towards vaccines and emotional orientations. This finding echoes the limitation of using sentiment analysis for investigating public attitudes to some extent. Sentiment analysis requires the consideration of contextual semantic understanding and domain knowledge that some utterances with different polarities may produce a specific emotional polarity in the text when placed together, and a collection of utterances without polarities may contribute to an intensive sentiment polarity [39]. For example, “In addition to the infection, the harm to our body after infection is also an important indicator, right? It would be a relief that immunization can make the impact of the COVID-19 on our health similar to that of the common cold. I do not want to face more individual tragedies.” Sentiment analysis tools judged the words “harm” and “tragedies” in this statement as negative polarities and sadness, but the post expressed a positive attitude towards COVID-19 vaccines. We further interpreted the content of posts with negative emotions and sentiments, discovering that the negative emotions and sentiments were related to distrust in the media and dissatisfaction with anti-vaccine information.

Results showed that sharp emotions and sentiments are directed to the media, posting users, and the public. Based on the content of these posts, the negative emotions and sentiments were mainly related to users’ dissatisfaction with Sina Weibo itself. They considered that the tag ‘most of the confirmed cases in Nanjing had been vaccinated’ would mislead people’s perception of COVID-19 vaccine effectiveness. A public perception that ‘vaccination is not effective’ would be created since the audience would believe that they still might be infected even if being vaccinated. Many users utilized sarcastic and contemptuous words to express negative emotions to reflect their disgust with Sina Weibo. In addition, they expressed their anger at the platform in a tone of questioning and accountability. Research on public opinions of social media users in Europe and the US also demonstrated that negative attitudes and hesitancy towards vaccines might be derived from external sources outside vaccines. The sources causing vaccine hesitancy might come from a lack of information and distrust in governments, doctors, medical institutions, and media [52,53]. Trust is also an influential factor in making information sources reliable and reducing the effects of disinformation [54].

The negative emotions and sentiments of the public were mainly reflected in their dissatisfaction with those who questioned the vaccine’s effectiveness. After the outbreak in Nanjing, some people began to doubt the vaccine’s efficacy, even questioning the development of the domestic COVID-19 vaccine. In response, other users viewed these people as having low literacy and low intelligence, posting offensive remarks such as ‘brainless’ and ‘uneducated’. They condemned people who questioned the domestic vaccine, arguing that ‘we should cherish the free vaccine developed at great expense’. They also used words with intense emotions such as ‘take the vaccine out of your body’ and ‘get out of China’. People who targeted their negative emotions at the posting users regarded them as having low comprehension and being non-assertive. They also pointed out that some posting users freely voiced their negative opinions of the vaccine expressed by online media, even without any knowledge of the epidemic and the vaccine.

Overall, the content of posts indicated that the expression of negative emotions and sentiments reflected an objective attitude towards the vaccines, as evidenced by the fact that 415 (59.63%) of 1025 posts with negative emotions were pro-vaccination, these posts showed an objective attitude of Weibo users towards the COVID-19 vaccine. These posting users are active social media consumers who actively follow and obtain information and express their opinions [55]. They had more information and knowledge about the vaccine, disagreed with the ‘one-sided’ media expression, and expected the media and the public to evaluate the vaccine from a scientific perspective. In other words, the expression of negative emotions reflected that active social media users defended the scientific communication of vaccines.

However, some posts were related to the direct venting of negative emotions rather than the discussion of vaccine effectiveness. This anonymous venting of emotions made the online space appear irrational. Research has showed that emotional expressions on social media are consistent with online self-presentation, and negative emotional expressions in an online space are more acceptable than in reality [56]. So, social media such as Sina Weibo can easily become a channel for the expression of negative emotions.

Previous research also proved that objective and well-documented health communication could increase persuasiveness [57]. A relevant question is how can health issues be communicated online objectively and effectively? One of the solutions is to build public trust in the media. On the one hand, the media needs scientific agenda-setting to avoid misleading topics. On the other hand, the press should guide the online public opinions and emotions with valuable and targeted information when the audience misunderstands the topic or vents negative emotions wildly, as health issues, especially COVID-19, are directly related to people’s safety and health, affecting the survival and development of the entire human population. The media should disseminate associated issues and promote people’s health behaviors via scientific and effective communication.

### 4.3. Unique Cultural Characteristics Shown in the Expression of Vaccine Attitudes by Chinese Social Media Users

As mentioned above, the attitudes of the Chinese public expressed on social media showed differences with those expressed by US Twitter users, and cultural differences between the two countries can explain the disparities.

On the one hand, the concerns with vaccines are different, comparing between China and Western countries. A study found significantly different content features of viral posts on Weibo and Twitter, reflecting the vast differences in perceptions of COVID-19 between the two major socio-cultural systems. Weibo users discussed COVID-19 as a public health issue rather than a societal and political issue, as it is discussed in Western societies. In general, Twitter users (mostly Western users) were highly engaged in discussions of responses, politics, and policies related to COVID-19. In contrast, Weibo users (most of whom were Chinese) were more inclined to focus on the virus and epidemic, but not exclusively [58]. Furthermore, the specific concerns about the pandemic indicated different value orientations. For instance, Twitter users were more concerned with economic health, whereas Weibo users cared more about public health. The discussion on Weibo had a prominent ‘scientific’ focus, emphasizing controlling the pandemic via scientific research methods, such as vaccine development and scientific experiments. However, Twitter users expressed more concerns about foreign affairs, trade policy, social employment, and economic status [37], which is consistent with our findings.

On the other hand, the Chinese public presented a collectivist cultural identity, unlike the individualistic cultural traits of the US. A comparison study in the social media discourse between China and the US revealed significant cultural differences between the two countries and platforms. The US users widely targeted government and politics, but Chinese users rarely targeted the same issues. Compared to US Twitter users, China’s Weibo users reflected more on firestorms that cause damage to social responsibility or the morally relevant dimensions of their targets [59]. Our research also verified this, showing that negative emotions expressed in the discussion about the Nanjing breakthrough cases were largely directed towards the social responsibility of the media. Moreover, positive emotions were directed to supporting the government, a cultural expression of collectivism among Chinese social media users. This particular argument supported Sethi’s finding that corporate social responsibility is closely associated with collectivist rather than individualist values, and Asian countries, such as China, are more inclined to express corporate social responsibility [60].

Another study examining the COVID-19-related discourse on Weibo and Twitter through a psycholinguistic analysis also validated the cultural differences in posts on social media [61]. Weibo users preferred using “we” more than Italian Twitter users using “I” [61]. For example, “Given the current situation of the pandemic internationally and the great advantages of vaccines in preventing severe infections, we should focus on promoting vaccination to protect our people from being seriously ill.” The increased use of “we” suggested that people are sharing more of a group identity [62], reflecting a collectivist culture in China.

Chinese Weibo users and Western Twitter users showed differences in their expression of positive orientations (such as trust in governments and acceptance of vaccines) and negative orientations (such as dissatisfaction with the media). The differences also indicated the disparities in the real world outside the social media sphere, such as cultural background, social value, health communication, and administration systems [37], providing a perspective for our further research.

## 5. Conclusions

This study suggested that online public opinion monitoring is an effective channel to track public opinions. However, sentiment analysis tools, which are commonly used methods, have limitations in investigating public attitudes towards vaccines and require more in-depth analysis to obtain reliable results. This study revealed intriguing findings through further content analysis—external sources outside of the vaccine might cause vaccine hesitancy, such as distrust in the media.

In addition, this study has certain limitations. We only monitor public opinions under one topic, not covering all public opinions. Furthermore, the voices of Sina Weibo users only show the views of active users and do not represent all types of attitudes among the public. We will conduct surveys and in-depth interviews to expand our research and further explore the influence of communication on public perceptions and attitudes towards vaccines.

## Figures and Tables

**Table 1:** Examples of sentiment words and sentiment coding of comments.

Items	Sample Words	Sample Sentences
Good	Trust, reliable, understand, generally accepted, sincerely, advancing, qualified, authoritative, pray, confidence	Scientists have been very hardworking and there will find a solution. We have to believe in the country, believe in science!
Happy	Convenience, reputation, pleasure, skill, smile, reliability, ease, fun, excitement, expectation	Vaccination can avoid severe illness, fortunately I did, ha ha ha ha!
Surprise	Strange, miraculous, sudden, rare, faint, occurring, up, shocking, startling, extreme	It’s really strange that vaccination still doesn’t prevent infection, only “no severe illness”.
Disgust	Stupidity, cunning, lies, exaggeration, shameful, rubbish, cursing, hypocrisy, filth, narrow-mindedness	So stupid people! The government gives you free vaccination and you still denigrated our country! You should quit our Chinese nationality then!
Sadness	Helplessness, pain, sadness, crying, melancholy, disaster, sting, guilt, failure, missing	The endless mutation, it feels like humans will be living in symbiosis with this virus. I miss the old days and can never go back.
Fear	Disease, panic, fear, ineffectiveness, convulsions, ills, bewilderment, narrowness, drastic changes, critical	The majority of confirmed cases have actually been vaccinated … Oh no, I’m scared.
Anger	Anger, rant, liar, rage, reproach, pain, protest, stare, accusation, rubbish	The vast majority of confirmed cases in Nanjing have been vaccinated? What a rubbish topic! The media is trying to get attention.
Other	Words contains no obvious emotional meaning	It is also important to fight the virus and to be physically fit myself. I need to go to the gym more often to work out.

**Table 2:** Content analysis coding table.

Items	Encoding Rules	Remarks
Attitudes towards COVID-19 vaccines	Supportive Neutral Doubtful Undetermined	Analyzes how the samples discuss or evaluate the COVID-19 vaccine and ascertains the poster’s attitude towards the vaccine.
Sentiment polarity	Positive Neutral Negative	According to the polarity of emotions, the emotions expressed in the posts are roughly divided into three categories.
Sentiment attribution	Good Happy Surprise Disgust Sadness Fear Anger Other	According to the DLUT-emotion ontology, the emotions expressed in posts are further divided into eight categories.
The specific targets of different sentiment orientations	COVID-19 vaccines Epidemic/COVID-19 Media Government/State The public Posting user Undetermined	The object pointed to by the Sentiment polarity and Sentiment attributes expressed by the sample.

**Table 3:** Statistical table of coding results (including statistics of sentiment polarity and sentiment attribution pointing to specific targets).

Items				Encoding Rules			All		Total (%)
Attitudes to COVID-19 vaccines				1. Supportive			696		45.14%
				2. Neutral			200		12.97%
				3. Doubtful			112		7.26%
				4. Undetermined			534		34.63%
**The specific targets of different sentiment orientations**									
Items	Encoding rules	1. COVID-19 vaccines	2. Epidemic/COVID-19	3. Media	4. Government/State	5. The public	6. Posting user	7. Undetermined	Total (%)
Sentiment polarity	1. Positive	178	4	11	7	0	0	0	200 (12.97%)
	2. Neutral	150	22	21	8	58	5	53	317 (20.56%)
	3. Negative	69	24	517	15	118	257	25	1025 (66.47%)
Sentiment attribution	1. Good	151	2	12	7	0	0	0	172 (11.15%)
	2. Happy	0	1	0	0	1	0	0	2 (0.13%)
	3. Surprise	10	0	3	0	2	1	3	19 (1.23%)
	4. Disgust	10	3	334	3	66	189	4	609 (39.50%)
	5. Sadness	6	10	1	1	2	3	4	27 (1.75%)
	6. Fear	17	14	6	1	6	0	10	54 (3.50%)
	7. Anger	9	1	132	10	46	57	5	260 (16.86%)
	8. Other	194	19	61	8	53	12	52	399 (25.88%)
Total (%)		397	50	549		30	176	262	78
		(25.75%)	(3.24%)	(35.60%)		(1.95%)	(11.41%)	(16.99%)	(5.06%)

**Table 4:** Pearson correlation coefficient and Chi-squared analysis of emotional orientations and attitudes towards vaccines.

Pearson Correlation Coefficient (Emotional Orientations and Attitudes towards Vaccines)								
0.323 **								
Chi-Squared Analysis (Emotional Orientations and Attitudes towards Vaccines)								
Items		Attitudes towards Vaccines (%)				Total	χ^2^	*p*
		1. Supportive	2. Neutral	3. Doubtful	4. Undetermined			
Emotional orientations	1 Positive	184 (26.44)	10 (5.00)	0 (0.00)	6 (1.12)	200 (12.97)	374.835	0.000 **
	2 Neutral	97 (13.94)	111 (55.50)	29 (25.89)	80 (14.98)	317 (20.56)		
	3 Negative	415 (59.63)	79 (39.50)	83 (74.11)	448 (83.90)	1025 (66.47)		
Total		696	200	112	534	1542		

** *p* < 0.01.

## Data Availability

The data presented in this study are available on request from the corresponding author Lina Li, at lilina@shnu.edu.cn. The data are not publicly available in accordance with funding requirements and participant privacy.
